# Comparison of the intima‐media thickness of the common carotid artery in patients with rheumatoid arthritis: A single‐center cross‐sectional case‐control study, and a brief review of the literature

**DOI:** 10.1002/hsr2.1718

**Published:** 2023-11-16

**Authors:** Farnood Rajabzadeh, Iman Akhlaghipour, Sahar Sadat Moosavi, Arya Nasimi Shad, Atefeh Babazadeh Baghan, Zhaleh Shariati‐Sarabi, Asma Payandeh, Ehsan Hassan Nejad

**Affiliations:** ^1^ Department of Radiology, Faculty of Medicine, Mashhad Medical Sciences Islamic Azad University Mashhad Iran; ^2^ Student Research Committee, Faculty of Medicine Mashhad University of Medical Sciences Mashhad Iran; ^3^ Mashhad Medical Sciences Islamic Azad University Mashhad Iran; ^4^ Rheumatic Diseases Research Center Mashhad University of Medical Sciences Mashhad Iran; ^5^ Faculty of Medicine Mashhad University of Medical Sciences Mashhad Iran; ^6^ Department of Radiology, School of Medicine Birjand University of Medical Sciences Birjand Iran

**Keywords:** atherosclerosis, carotid intima‐media thickness, rheumatoid arthritis, ultrasonography

## Abstract

**Background and Aim:**

Rheumatoid arthritis (RA) is an autoimmune chronic inflammatory disease affecting 0.5%−1% of adults worldwide. The carotid intima‐media thickness (CIMT) is a simple, reliable, noninvasive marker for subclinical atherosclerosis. The aim of this study was to compare the intima‐media thickness of the common carotid artery in patients with RA with that of healthy patients.

**Methods:**

In this case‐control study, subjects were recruited from the patients who presented to a private rheumatology clinic. RA was documented by a rheumatologist. All subjects underwent an ultrasound examination of the carotid artery to assess CIMT. Subjects with RA filled out the disease activity score (DAS28) questionnaire.

**Results:**

Sixty‐two subjects (31 subjects with RA and 31 healthy subjects) took part in the study. The mean age of the subjects in the RA and the control groups was 42.39 ± 12.98 and 44.48 ± 13.56 years, respectively. Values of CIMT were significantly greater in RA subjects compared with their healthy counterparts (*p* < 0.001). The CIMT increased significantly with increased disease severity (*r* = 0.73). Subjects were divided into two age groups (≤40 and >40 years). A comparison of CIMT in the mentioned subgroups revealed a remarkable difference in CIMT values between those of the RA patients and those of their control counterparts in both age groups (*p* = 0.002 and *p* < 0.001 for those below and above 40 years, respectively).

**Conclusion:**

CIMT could be used as an efficient clinical index for identifying the early stages of atherosclerosis and predicting cardiovascular events following atherosclerosis in RA patients.

## 1 INTRODUCTION

Rheumatoid arthritis (RA) is a chronic autoimmune disease that affects various body organs and has systematic manifestations such as cardiovascular, neurological, lung, and skin involvement.^1^, ^2^ RA affects nearly 0.5%−1% of adults globally and affects women more than men.^3^ The underlying cause of RA is unknown and is defined by symmetric peripheral polyarthritis.^4^ Mortality rates is higher in RA patients than in the healthy population, and cardiovascular disease (CVD) is the most common cause of mortality in RA patients.^1^, ^5^, ^6^ The reason for the increased risk of CVD in RA patients is multiple, which includes the increasing prevalence of traditional CVD risk factors (such as hypertension and dyslipidemia), drug‐related risk factors, as well as the persistent inflammatory load associated with RA, and their consequences, such as vascular dysfunction.^2^, ^7^ However, It was estimated that systemic inflammation was the main contributor to atherosclerosis in RA patients.^6^, ^8^, ^9^ The onset of atherosclerosis is due to dysfunction of the endothelium, in which damage to the thin wall of the inner surface of blood vessels causes the accumulation of fatty streaks in the artery wall.^10^ Most of the specific risk factors for RA, including the inflammatory biomarkers and anti cyclic citrullinated peptide (anti‐CCP), were found to be related to increased carotid intima‐media thickness (CIMT) and increased risk for CVD. CIMT is the thickness of the carotid artery wall that is measured by B‐mode ultrasonography.^6^, ^8^ Measuring the distance between lumen‐Intima and media‐adventitia is called CIMT^10^

CIMT is considered an early surrogate marker for atherosclerosis.^5^ CIMT is a noninvasive, cheap, and reliable marker for subclinical atherosclerosis. Increased CIMT has been reported in the early stages of atherosclerosis in various studies in the general population and among patients with RA.^11^, ^12^, ^13^, ^14^, ^15^, ^16^, ^17^ Because of the increased risk for CVD in patients with RA, using ultrasound to assess the CIMT in patients with RA is promising. The aim of this study was to compare the CIMT of RA patients with that of non‐RA subjects. Our study not only entailed a comparison of CIMT between individuals with RA and those without, but also involved an assessment of RA severity through a questionnaire, followed by an evaluation of any potential correlation between CIMT and RA disease severity. We also investigated the potential increase of CIMT among RA patients as they age. Besides CIMT, we assessed the PSV, RI, PI, and EDV of the common carotid artery and compared these variables between RA and non‐RA individuals.

## 2 METHODS

In this cross‐sectional case‐control study, subjects with RA were selected from among the patients who were referred to a private rheumatology clinic, while the unmatched control group was chosen from the population of non‐RA patients who were referred to the clinic. All patients who were older than 16 years were included in the study. Subjects were excluded if they had a body mass index > 30 kg/m^2^, were active smokers, had a positive history of CVD, heart failure, hypertension, or had documented diabetes mellitus or chronic renal failure (glomerular filtration rate <60). A rheumatologist made the diagnosis of RA. Subjects were recruited based on convenience sampling. Before participation, each subject signed written informed consent. The ethical committee of the Azad University of Mashhad approved this study.

The sample size was calculated based on the sample size formula for case‐control studies suggested by Kesley et al. with the power of 90 and confidence interval (CI) = 99%.^18^ The sample size was calculated to hold 18 subjects in each study group. Considering a 20% dropout, the sample size was increased to 22, and assuming a 40% effect size, the final sample size was calculated to consist of 31 subjects in each study group.

All subjects underwent ultrasound scans to assess the CIMT at 5, 10, and 15 mm below the bifurcation of the common carotid with the General Electric (GE) Logiq 500 unit (General Electric Medical Systems) using a 9−7 MHz linear transducer in the supine position and a 45° head tilt. The values of CIMT, peak systolic velocity (PSV), resistance index (RI), pulsatility index (PI), and end‐diastolic velocity (EDV) were recorded for all subjects.

Disease severity was assessed in RA patients using the disease activity score 28 (DAS28) questionnaire. This questionnaire comprises eight items, and the score can range from 0 to 10. DAS28 scores below or equal to 3.2 are considered low disease activity, scores between 3.3 and 5.1 are categorized as moderate, and values greater than 5.1 are regarded as severe disease activity.^19^

### 2.1 Statistical analysis

The statistical analysis was conducted apby plying the statistical package for social sciences (SPSS) version 21 (IBM Inc.) and Statistica version 10 (StatSoft Inc.). The normality of the variables was assessed using the Shapiro−Wilk test. Mean and standard deviation were used to describe continuous variables. To compare variables between groups, Student's *t*‐test and analysis of variance were applied. All statistical tests were applied two‐sided. Frequency and percentage were used to describe categorical values. The *χ* ^2^ test was used to compare the distribution pattern of categorical variables between study groups. Using the linear/logistic regression and while controlling for age and gender, we assessed the relationship between CIMT and age. The significance level was considered 0.05, and the CI was considered 0.95.

## 3 RESULTS

Sixty‐two subjects (36 females and 26 males), consisting of 31 subjects in each group, took part in this study. The mean age of the subjects in the RA and the control groups was 42.39 ± 12.98 and 44.48 ± 13.56 years, respectively. In the RA group, 19 subjects (61.3%) were female, and 12 (38.7%) were male, while in the control group, 17 subjects (54.8%) were female, and 14 (45.2%) were male.

The mean CIMT, PSV, RI, PI, and EDV values in the study groups are shown in Table 1. Except for the latter, the results for all other factors showed a meaningful difference between the two groups.

**Table 1:** Comparison of study parameters as per study groups.

Value	Group		*p* Value
	Mean ± SD		
	RA	Control	
CIMT	0.68 ± 0.17	0.48 ± 0.07	<0.001*
PSV	73.13 ± 19.40	85.45 ± 20.42	0.02*
RI	0.69 ± 0.07	0.75 ± 0.09	0.01*
PI	2.53 ± 1.13	1.79 ± 0.44	0.01*
EDV	21.69 ± 7.89	20.20 ± 4.39	0.37

Abbreviations: CIMT, carotid intima‐media thickness; EDV, end‐diastolic velocity; PI, pulsatility index; PSV, peak systolic velocity; RA, rheumatoid arthritis; RI, resistance index; SD, standard deviation.

* significant level = *p*‐value <0.05.

There was a meaningful difference in CIMT values between the severity categories of RA (*p* < 0.001) (Table 2). The Spearman's correlation coefficient also revealed a significant relationship between RA severity and CIMT values, showing higher CIMT values with a more severe RA disease activity (*r* = 0.73, *p* < 0.05).

**Table 2:** Comparison of CIMT values between RA severity categories.

RA severity	CIMT mean ± SD	*p* Value
Mild	0.53 ± 0.07	<0.001*
Moderate	0.62 ± 0.11	
Severe	0.84 ± 0.14	

Abbreviations: CIMT, carotid intima‐media thickness; RA, rheumatoid arthritis; SD, standard deviation.

* significant level = *p*‐value <0.05.

The study subjects were divided into two age groups (≤40 and >40 years). As illustrated in Table 3, a comparison of CIMT in the mentioned subgroups revealed a remarkable difference in CIMT values between those of the RA patients and those of their control counterparts in both age groups (*p* = 0.002 and *p* < 0.001 for those below and above 40 years, respectively).

**Table 3:** Comparison of CIMT values between study groups as per age categories.

Group	RA mean ± SD	Control mean ± SD	*p* Value
≤40 years	0.53 ± 0.07	0.45 ± 0.03	0.002*
>40 years	0.75 ± 0.15	0.54 ± 0.09	<0.001*

Abbreviations: CIMT, carotid intima‐media thickness; RA, rheumatoid arthritis; SD, standard deviation.

* significant level = *p*‐value <0.05.

Five (16.13%) of the 31 subjects in the RA group had carotid plaques, while this figure was 4 (12.9%) in the control group. There was no difference in the distribution pattern of carotid plaques between the two groups (*χ* ^2^ = 0.13, *p* = 0.72).

## 4 DISCUSSION

CVD is one of the leading causes of morbidity and mortality in RA patients^20^, ^21^, ^22^ It has been shown that the rate of CVD‐related mortality in RA patients increases by 50% compared to the general population.^23^ CIMT can be used as an important factor for future cardiovascular events and as a sign of early atherosclerosis.^23^, ^24^

This study revealed that, in both the RA and control groups, the CIMT value was higher in those over 40 years of age than in their younger counterparts. This finding was in line with results obtained by Jayakumar et al., who revealed higher CIMT values in Indian RA patients who were between 41 and 55 years old^25^ and with results presented in other studies that reported increased CIMT in RA patients with aging^5^, ^26^, ^27^

This study also revealed that CIMT increased significantly with increased disease severity in RA. Previous studies have also identified increased CIMT with worsening disease states in RA patients.^5^, ^26^ These studies also revealed that the CIMT values increased with the RA duration, a finding not assessed in the current study.^5^, ^26^ As with our findings regarding disease severity got through the DAS28 questionnaire, another study observed a significant increase in ESR and CRP levels in RA patients with higher CIMT values.^28^

This study revealed higher CIMT in RA patients in all age groups than the values obtained from the control group. This was in line with the findings of previous research.^1^ However, not all studies found meaningful differences in CIMT between RA and the control subjects.^26^ This lack of difference might have been due to including RA patients with mild disease activity. The incidence of atherosclerotic plaques in our RA population was 16.13%, which, however insignificant, was slightly higher than the previously reported figure of 15.6% by Mohan et al.^1^ It was previously reported that atherosclerotic plaques were significantly more prevalent in RA patients than amongst the control group.^29^

Table 4 shows a summary of the results from the other studies.

**Table 4:** Summary of the results from the other studies.

The first author (reference)	RA patients			Controls			CIMT		
	*N*	Age (years)	Female, *n* (%)	*N*	Age (years)	Female, *n* (%)	RA patients	Controls	*p* Value
		Mean ± SD			Mean ± SD		Mean ± SD	Mean ± SD	
Tuzcu et al.^30^	39	47.7 ± 8.8	6 (15.4)	30	43.0 ± 10.8	9 (30)	0.63 ± 0.17	0.51 ± 0.12	0.001
Biskup et al.^31^	70	53.9 ± 13.1	54 (77.1)	33	53.6 ± 8.3	18 (54.5)	0.83 ± 0.21	0.62 ± 0.1	<0.001
Gauri et al.^32^	50	46.56 ± 12.82	41 (82)	50	46.38 ± 11.69	NA	0.5996 ± 0.109	0.5290 ± 0.006	<0.001
Ulsu et al.^33^	52	46.55 ± 9.03	41 (78.7)	46	43.69 ± 8.87	28 (60.9)	0.80 ± 0.22	0.51 ± 0.18	<0.0001
Kim et al.^12^	44	59.98 ± 9.34	44 (100)	22	57.95 ± 6.87	22 (100)	0.705 ± 0.198	0.611 ± 0.093	<0.05
Kisiel et al.^34^	317	57.61 ± 12.62	259 (81.70)	111	55.50 ± (9.37)	89 (80.18)	0.718 ± 0.181	0.682 ± 0.167	0.07
Elshereef et al.^35^	112	36.50 ± 7.22	94 (83.92)	40	34.6 ± 8.22	32 (84.2)	0.057 ± 0.016	0.050 ± 0.007	0.018
Lo Gullo et al.^36^	33	49.6 ± 7.6	19 (57.57)	33	47.4 ± 6.4	19 (57.57)	1.16 ± 0.2	0.81 ± 0.1	<0.001
Im et al.^37^	406	55 ± 12.3	328 (80.8)	209	55 ± 12.1	169 (80.9)	0.792 ± 0.169	0.714 ± 0.136	<0.001
Adhikari et al.^38^	35	38.3 ± 10.6	29 (82.85)	35	36.1 ± 8.2	28 (80)	0.50 ± 0.16	0.44 ± 0.09	0.007

Abbreviations: CIMT, carotid intima‐media thickness; RA, rheumatoid arthritis; SD, standard deviation.

Considering the observed higher values of CIMT in RA patients compared to those of the controls, this study suggests CIMT assessment as a sensitive marker in assessing the risk of CVD in patients with RA.

It is recommended that further studies with greater sample sizes be performed to assess better the relation between CIMT and CVD risk in RA patients. It is also suggested that disease duration be taken into consideration.

## AUTHOR CONTRIBUTIONS

**Farnood Rajabzadeh**: Conceptualization; data curation; project administration; visualization; writing—review and editing. **Iman Akhlaghipour**: Data curation; formal analysis; methodology; project administration; visualization; writing—original draft; writing—review and editing. **Sahar Sadat moosavi**: Data curation; writing—original draft; writing—review and editing. **Arya Nasimi Shad**: Formal analysis; writing—original draft. **Atefeh Babazadeh Baghan**: Formal analysis; writing—original draft. **Zhaleh Shariati‐Sarabi**: Project administration; writing—review and editing. **Asma Payandeh**: Data curation; methodology; writing—original draft; writing—review and editing. **Ehsan Hassan Nejad**: Data curation; project administration; visualization; writing—original draft; writing—review and editing. All authors have read and approved the final version of the manuscript.

## CONFLICT OF INTEREST STATEMENT

The authors declare no conflict of interest.

## ETHICS STATEMENT

The method was approved in terms of compliance with scientific and ethical standards. All methods were performed in line with the relevant guidelines and regulations. The Medical Ethics Committee of Mashhad Medical Science Islamic Azad University approved this study and all patients and their families were informed and signed the informed consent.

## TRANSPARENCY STATEMENT

The lead author Ehsan Hassan Nejad affirms that this manuscript is an honest, accurate, and transparent account of the study being reported; that no important aspects of the study have been omitted; and that any discrepancies from the study as planned (and, if relevant, registered) have been explained.

## Data Availability

The data sets created during the current study are not publicly accessible due to the possibility of compromising the privacy of individuals. Ehsan Hassannezhad had full access to all of the data in this study and takes complete responsibility for the integrity of the data and the accuracy of the data analysis.
